# Olfaction and Sarcopenia in Older Adults: A Longitudinal Study

**DOI:** 10.1093/geroni/igaf122.2966

**Published:** 2025-12-31

**Authors:** Rui Liu, Honglei Chen, Chenxi Li, Anna Kucharska-Newton, Eleanor Simonsick, Yaqun Yuan

**Affiliations:** Sacred Heart University, Fairfield, Connecticut, United States; Michigan State University, East Lansing, Michigan, United States; Michigan State University, East Lansing, Michigan, United States; The University of North Carolina at Chapel Hill, Chapel Hill, North Carolina, United States; National Institute on Aging, Baltimore, Maryland, United States; Michigan State University, East Lansing, Michigan, United States

## Abstract

Olfactory impairment is associated with greater weight loss in older adults. We examined olfaction in relation to sarcopenia in 2503 participants (aged 71-82, 51.7% women, and 38.4% Black) of the Health, Aging, and Body Composition study. Olfaction was assessed in Year 3 using the 12-item Brief Smell Identification Test, categorized as good (test score: 11-12), moderate (9-10), hyposmia (7-8), and anosmia (0-6). We measured sarcopenia as slow gait speed (20-meter usual walking speed < 0.8 meters/second) or low muscle strength (grip strength < 35.5 kg for men and < 20 kg for women), which were assessed annually/biennially for up to 7 years. We conducted joint model analyses to account for covariates and potential bias due to attrition. Compared with participants with good olfaction, the multivariable adjusted odds ratio (OR) and 95% confidence interval (CI) associated with slow gait speed at baseline was 1.05(0.86, 1.29) for moderate olfaction, 1.13(0.87, 1.46) for hyposmia, and 1.98(1.50, 2.61) for anosmia. Notably, associations strengthened over time. At the end of follow-up, corresponding ORs increased to 1.80(1.17, 2.76) for moderate olfaction, 2.68(1.54, 4.67) for hyposmia and 3.74(2.09, 6.71) for anosmia. For muscle strength, we only found an association with anosmia but not hyposmia or moderate olfaction. At the end of the follow up, compared to those with good olfaction, the ORs for low muscle strength were 1.32(0.80, 2.19) for moderate olfaction, 1.19(0.63, 2.24) for hyposmia, and 3.12(1.48, 6.55) for anosmia. Poor olfaction was associated with markers of sarcopenia in older adults, particularly reduced mobility.

